# Berberine in Sepsis: Effects, Mechanisms, and Therapeutic Strategies

**DOI:** 10.1155/2023/4452414

**Published:** 2023-01-25

**Authors:** Tingxia Lv, Chunpan Zhang, Lan Hu, Chao Wang, Shirong Li, He Wang, Wenjie Qi

**Affiliations:** Department of Infectious Diseases, Beijing Friendship Hospital, Capital Medical University, Beijing 100050, China

## Abstract

Sepsis is defined as a dysregulated immune response to infection that leads to multiple organ dysfunction. To date, though a growing body of knowledge has gained insight into the clinical risk factors, pathobiology, treatment response, and recovery methods, sepsis remains a significant concern and clinical burden. Therefore, further study is urgently needed to alleviate the acute and chronic outcomes. Berberine (BBR), a traditional Chinese medicine with multiple actions and mechanisms, has been investigated in cellular and rodent animal models of sepsis mainly based on its anti-inflammatory effect. However, the practical application of BBR in sepsis is still lacking, and it is imperative to systematically summarize the study of BBR in sepsis. This review summarized its pharmacological activities and mechanisms in septic-related organ injuries and the potential BBR-based therapeutic strategies for sepsis, which will provide comprehensive references for scientific research and clinical application.

## 1. Introduction

Sepsis is a life-threatening clinical syndrome characterized by multiple organ dysfunction caused by the body's dysregulated response to infection. Despite better supportive therapy and care, sepsis and sepsis shock have led to a high morbidity and mortality rate in recent years [1]. Just as intricated immune or inflammatory factors drive multiple metabolic diseases [2, 3], the exact mechanisms responsible for sepsis are also complex, involving coagulation abnormalities, uncontrolled release of inflammatory mediators, excessive innate immune response, endothelial capillary leakage syndrome, and organ dysfunction [4]. Though progress has been made regarding recognizing and managing clinical sepsis, incidence and mortality rates remain high. For example, in 2017, an estimated 48.9 million incident cases of sepsis and 11.0 million sepsis-related deaths were reported, representing 19.7% of all global deaths [5, 6]. Furthermore, clinical trials of therapeutics have failed to obtain promising results, and it likely got even worse during the coronavirus disease 2019 (COVID-19) pandemic due to the limited intensive care unit resources [7]. Given the lack of effective treatment for sepsis, Chinese herbal medicines, i.e., Chinese patent medicines, Chinese herbal prescriptions, and single Chinese herbs, have therapeutic actions promising in treating sepsis through multicomponent, multipathway, and multitargeting abilities [8].

Berberine (BBR), an isoquinoline alkaloid, is widely distributed in many medicinal plants, such as *Coptis chinensis* and *Berberis vulgaris*, and is usually regarded as an ingredient of Chinese herbal medicines [9]. It has a molecular weight of 336.37 Da and can be obtained through de novo synthesis. Though BBR was approved to be an antibacterial agent, it also plays a crucial therapeutic role in infectious diseases, cardiovascular diseases, neurodegenerative diseases, and rheumatoid arthritis due to its antimicrobial, antioxidant, anti-inflammatory, and metabolic regulation effects [10, 11]. BBR can act on a diverse range of molecular targets by binding to the active cavities with specific structural and physiochemical properties. Among the related conditions, sepsis is characterized by the host's excessive and complex inflammatory response and is linked with bacterial and viral infections during its occurrence and progression. In recent years, though no studies about BBR for treating sepsis in clinical practice, its protective effect and mechanism on sepsis have been explored extensively in cellular and animal models [12–15]. Whether BBR could be a potential therapeutic drug for sepsis and septic complications remains unclear in the real world. Given that dysregulated host systemic inflammatory and immune response during sepsis usually lead to life-threatening organ dysfunction, this paper summarizes the protective role of BBR for organ injury and related mechanism and, finally, prospects the potential BBR-based therapeutic strategies in sepsis.

## 2. Effects and Mechanisms of BBR in Sepsis-Related Organ Dysfunction

### 2.1. BBR in Sepsis-Related Intestinal Barrier Injury

The intestinal barrier and the subsequent translocation of intestinal bacteria play a critical role in multiple organ dysfunction syndromes during sepsis [16]. Lipopolysaccharide (LPS) produced by intestinal bacteria might damage gut-vascular integrity, increase permeability, and reduce tight junction and adherens junction protein production [17]. Several studies have shown that pretreatment with BBR protects against intestinal injury in sepsis-challenged animals with various mechanisms. For example, in male Sprague-Dawley (SD) rats undergoing cecal ligation and puncture (CLP), TNF-*α* and IL-6 were significantly lower, while the tight junction protein level, the percentage of cell death in intestinal epithelial cells, and the mucosal permeability were significantly elevated after treatment by BBR [12]. The overall effects were consistent with other studies [18, 19]. Further investigation showed that the mRNA level of intestinal Toll-like receptors 2 (TLR2) and TLR4 was also increased [18, 20], indicating that BBR might protect the intestinal mucosal barrier in the early phase of sepsis through the TLR-NF-*κ*B signaling pathway [20]. Moreover, BBR protected the damaged gut-vascular barrier (GVB) via modulation of the hepatic apolipoprotein M/S1P pathway in the polymicrobial sepsis model [21] and attenuated the impairment of glutamine transport and glutaminase activity in rats [19]. BBR also inhibited inducible cyclooxygenase-2 (COX-2) overexpression in rat small intestinal mucosa via activation of the peroxisome proliferator-activated receptor-gamma (PPAR*γ*) pathway and inhibited the effects of LPS in a rat model of endotoxemia [13]. However, although NOD-like receptors (NLR) play a crucial role in host defense against intestinal infection, there is no evidence that the NLR pathway was related to the preventive effect of BBR for sepsis [12]. In contrast, Wnt/beta-catenin signaling pathway was related to the protective effect of BBR on the gut-vascular barrier during sepsis [17].

Zinc deficiency is prevalent in the gut and might drive bacterial translocation and toxin dissemination, while zinc redistribution could protect the intestinal barrier in sepsis [22–24]. It was reported that BBR could regulate the trace elements of zinc's metabolism and exert intestinal protection role. He et al. demonstrated that BBR (50 mg/kg/day, gavage) improved survival rates of septic rats in the rat CLP sepsis model (BBR vs. CLP group: 80% vs. 46.7% at 48 hours) and reduced plasma endotoxin levels and intestinal mucosal permeability of septic rats, which were accompanied by the increased zinc levels [25]. In cellular studies, BBR treatment increased intracellular zinc transporter Zrt-Irt-like protein 14 (ZIP14) mRNA and protein expression in LPS-treated human colorectal adenocarcinoma cell line Caco-2 cells and upregulated LPS-decreased claudin-1 and occludin generation [25]. From the above evidence, we could conclude that BBR has a definite protective effect on intestinal barrier dysfunction, and this aspect might be the first breakthrough of BBR in treating organ injury in sepsis.

### 2.2. BBR in Sepsis-Related Lung Injury

Lung injury and acute respiratory distress syndrome (ARDS) are common challenges in sepsis. Microbial infections are usually responsible for pneumonia or sepsis, which cause altered respiratory function, pulmonary edema, and accumulation of inflammatory cells, including polymorphonuclear neutrophils (PMNs), circulating monocytes, and tissue-resident macrophages. The protective role of BBR for lung injury in sepsis was mainly derived from its anti-inflammatory effect. BBR was endowed with pronounced anti-inflammatory property, which was probably associated with suppressing the activation of the NF-*κ*B signaling pathway and the subsequent gene expressions and productions of proinflammatory mediators [26, 27], as well as suppressing the phosphorylation of MAPKs, such as p38, ERK, and JNK, and the level of reactive oxygen species in macrophages [28]. Berberine pretreatment maintained the integrity of endothelial glycocalyx, inhibited NF-*κ*B signaling pathway activation, and decreased the production of proinflammatory cytokines TNF-*α*, IL-1*β*, and IL-6 in mice with LPS-induced ARDS [29]. Pretreatment with neutral sulfate berberine attenuates lung and small intestine tissue injury and improves survival in endotoxemic mice, which may be mediated, at least in part, by the inhibition of proinflammatory mediators TNF-*α* and IFN-*γ* and NO production and upregulation of IL-10 release [1]. Wang et al. also reported that BBR inhibited LPS or CpG-ODN-induced proinflammatory cytokine expression at 12 and 24 h post BBR treatment in bone marrow-derived macrophages (BMDM) and LPS/D-galactosamine-challenged septic mouse model in vivo. They demonstrated that BBR significantly attenuated lung tissue injury and potently increased the survival rate in the septic mice with negligible side effects by suppressing NF-*κ*B- and IL-6-mediated STAT3 activation [30]. Additionally, BBR could protect the permeability of pulmonary microvascular endothelial cells (HPMECs) and decrease the expression of IL-1*β* and IL-18 by suppressing the expression of phosphorylated NF-*κ*B and further restraining the downstream gene nucleotide-binding domain and leucine-rich repeat protein-3 (Nlrp3) [31]. This limited evidence hints that the anti-inflammatory roles might be the core mechanisms for BBR in treating sepsis-related lung injury.

### 2.3. BBR in Sepsis-Related Acute Kidney Injury

Septic acute kidney injury (S-AKI), which is characterized by a rapid deterioration of renal function, accounts for about half of all patients with AKI syndrome treated in the intensive care unit (ICU) [32]. The mechanisms of sepsis-associated acute kidney injury involve ischemia-reperfusion, inflammation, oxidative stress, tubular cell damage, dysregulated microcirculation, morphofunctional alterations in the mitochondria, and apoptosis. The pathogen-associated molecular patterns (PAMP) and damage-associated molecular patterns (DAMP) participate in the pathological process [33, 34]. Sepsis-causing pathogens include bacteria, fungi, and viruses, and the widely used models of S-AKI in basic research were usually induced by bacteria or isolated bacterial pathogenic substances [33]. As an alkaloid with well antibacterial activity and even an alternative to antibiotics, few studies reported the application of BBR in S-AKI. Al-Kuraishy et al. revealed that BBR has no significant effect on AKI biomarkers except on serum kidney injury molecules (KIM-1) in diclofenac-induced AKI, whereas combined use of BBR and pentoxifylline significantly reduced renal biomarkers such as blood urea, serum creatinine, and glomerular filtration rate (eGFR) [35]. Berberine is the main bioactive component in *Rhizoma Coptidis* [36]. Integrating metabolomics and network pharmacology showed that *Rhizoma Coptidis extracts* increased the nuclear translocation of nuclear factor-erythroid 2-related factor-2 (Nrf2), the protein expression of heme oxygenase-1 (HO-1), and the mRNA expression of PPAR*α* and reduced nitric oxide synthase 2 (NOS2) activity [37]. Of note, BBR also exerts renoprotective effects caused by other inducers such as cisplatin and gentamicin in rodent models through upregulating mitophagy or its antioxidant, anti-inflammatory, and antiapoptotic properties [38–40].

### 2.4. BBR in Sepsis-Related Liver Injury

Disturbed liver function and drastic changes in hepatic gene and protein expression during sepsis induce inflammatory pathway activation and downregulate house-keeping functions such as metabolic, biotransformation, or bile transport activities [41]. During systemic infections, the liver regulates immune defenses via bacterial clearance, production of acute-phase proteins (APPs) and cytokines, and metabolic adaptation to inflammation. The release of APPs with various biological functions in the systemic circulation contributes to the systemic activation of immune responses, while mitochondrial and endoplasmic reticulum (ER) dysfunctions during the acute phase response elicited by systemic inflammation lead to liver failure in sepsis [3, 41, 42]. To date, the molecular mechanism studies for hepatoprotection of BBR in sepsis were insufficient. Limited investigations revealed that BBR inhibits LPS-induced oxidative stress markers NO protein and iNOS expression in RAW 264.7 and THP-1 macrophage cells [14] and further alleviates ALT, AST, malondialdehyde (MDA), and myeloperoxidase (MPO) activity in sepsis rat model [15]. Moreover, BBR reduced proapoptotic protein cleaved caspase-3 and increased antiapoptotic protein Bcl-2 in CLP-induced liver injury, which were associated with a reduction in intestinal permeability and CLP-induced distant organ cell apoptosis as compared with mice on vehicle treatment [43]. Among the numerous mediators in sepsis, the high mobility group box 1 (HMGB1) is an endogenous DAMP that mediates downstream effects within the inflammatory cascade. It was reported that AMPK activation could significantly inhibit LPS-induced HMGB1 release in RAW264.7 cells [44]. A berberine derivative, 13-ethylberberine (13-EBR), could reduce HMGB1 release by activating AMPK under septic conditions and protect endotoxemic mice from liver damage [45]. Similarly, other researchers also have verified the inhibition of BBR-loaded nanostructured lipid carriers for HMGB1/TLR4/NF-*κ*B signaling and protective roles for warm hepatic ischemia/reperfusion-induced lesion [46]. Therefore, based on this limited evidence, inhibiting the key inflammatory mediators and pathways is still a dominant mechanism of BBR in treating sepsis-related liver injury.

### 2.5. BBR in Sepsis-Induced Disseminated Intravascular Coagulation

Disseminated intravascular coagulation (DIC) was recognized as a deadly complication in sepsis. In addition to the activation of coagulation induced by pathogens, damage-associated molecular patterns, neutrophil extracellular traps, extracellular vesicles, and glycocalyx damage are also involved in the pathogenesis of sepsis-induced DIC. Tissue factor (TF) plays a crucial role in the coagulation of sepsis [47]. It was reported that BBR could inhibit LPS-induced TF activity and expression and downregulate NF-*κ*B, Akt, and MAPK/JNK/p38/ERK pathways in THP-1 cells, which provides some new insights into its mechanism for sepsis treatment [48]. Yang et al.'s study showed that caspase-11, as a cytosolic LPS receptor, could enhance the activation of tissue factor and subsequent phosphatidylserine exposure independent of cell death, while deletion of caspase-11 or neutralization of phosphatidylserine or TF prevented LPS-induced DIC [49]. Berberine inhibits caspase-11-dependent coagulation in bacterial sepsis by blocking Msr1, thus preventing coagulation syndrome [50]. Mechanistically, Wang et al. first reported the possible targets and mechanisms of BBR and its primary metabolite berberrubine in inhibiting platelet activation. That is, BBR significantly inhibited ADP-induced integrin *α*IIb*β*3 activation, reduced the level of P-selectin on the platelet membrane, and suppressed the binding of fibrinogen to the platelets, which was derived from the inhibition roles of BBR for the PI3K/Akt pathway, Rasa3 membrane translocation, and Rap1 activation [51]. These existing studies open a promising direction for BBR in treating sepsis-induced disseminated intravascular coagulation.

## 3. Berberine-Based Therapeutic Strategies in Sepsis

Although many studies have reported the protective effects and multiple mechanisms of BBR in sepsis-related animal or cell models (Table 1), unfortunately, there is no clinical practice at present. We speculated that to treat this kind of systemic severe inflammatory disease, it still needs to improve the drug concentration of BBR in blood, discover compounds with better efficacy based on the structure of BBR, and explore drug combination strategies in the future. Definitely, the poor oral bioavailability of BBR limited its therapeutic potential, and fortunately, BBR solid lipid nanoparticles (BBR-SLNs) were previously developed and showed significant improvement in bioavailability and reduction in systemic adverse reactions [52]. In recent years, BBR-SLNs have been applied for the management of ulcerative colitis [53, 54], bacterial infection [55], and infections of traumatic wounds [56], which are essential sepsis triggers. The effect of immunomodulatory activity and inhibition for the expression of multiple inflammatory factors were demonstrated, suggesting that BBR-SLNs might be developed to enhance the therapeutic effects of BBR as an antisepsis drug. Besides, several derivatives of BBR were reported to have better pharmaceutical effects. For example, the natural oxoderivative oxyberberine (OBB) was endowed with more pronounced anti-inflammatory properties than BBR, which was likely to be associated with suppressing the activation of the NF-*κ*B, the subsequent gene expressions and productions of proinflammatory mediators [26]. Tetrahydroberberrubine (THBru), a berberine derivative, significantly decreased TNF-*α* and nitrate/nitrite content in the plasma, reduced the MPO activity in LPS-induced acute lung injury, and suppressed the activation of the MAPKs, AKT, and the NF-*κ*B subunit p65 in LPS-induced THP-1 cells [57]. Similarly, another berberine derivative, 13-ethylberberine (13-EBR), reduces HMGB1 release through AMPK activation in LPS-activated RAW264.7 cells and protects endotoxemic mice from lung and liver damage [45]. Wang et al. considered that BBR might also serve as a promising adjuvant that could be used in conjunction with other medications for the treatment of septic patients [30]. For example, combinatorial liposomes of BBR and curcumin could inhibit biofilm formation and intracellular methicillin-resistant *Staphylococcus aureus* infections and associated inflammation, with five times more efficient than clindamycin [58]. The combined use of sodium new houttuyfonate and berberine chloride presented synergistic antibacterial effects against growing and persistent methicillin-resistant and vancomycin-intermediate *Staphylococcus aureus*, which is likely related to the interruption of the cell membrane [59]. These combined antibacterial effects of BBR with other agents predicted their potential application in sepsis. Taken together, the strategies for improving the bioavailability, discovering more powerful and effective berberine derivatives, and drug combinations might contribute to the application of BBR in treating sepsis in the future.

## 4. Conclusion and Perspective

**Figure 1:**
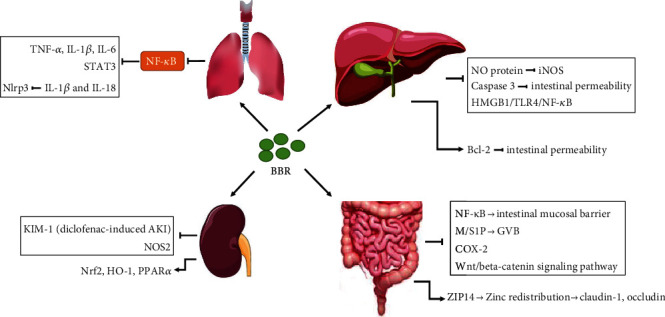
The protective effect and mechanism of berberine for sepsis-related organ injury.

BBR, as a natural modulator of inflammatory signaling in multiple immune and infectious diseases, could also effectively protect the various organs in sepsis from severe damage and increase the survival rate in rodent models. Currently, BBR could be considered to act as a multitarget drug to antagonize sepsis. The primary mechanism mainly derives from the regulation for inflammatory signaling pathways (i.e., NF-*κ*B, JAK/STAT, Akt, and MAPK), the key inflammatory mediators (i.e., HMGB1 and tissue factor), the subsequent anti-/proinflammatory cytokines (i.e., TNF-*α*, IL-6, and IL-10), and the dysregulated endoplasmic reticulum/oxidative stress and apoptosis (Figure 1). BBR exhibited potential therapeutic effects for sepsis-related intestinal barrier injury, lung injury, acute kidney injury, liver injury, and disseminated intravascular coagulation. Given the exact antibacterial effect of BBR and a large number of experimental reports in intestinal regulation, it is expected to be first used to protect against sepsis-induced intestinal injury in the clinic. Also, owing to its poor oral bioavailability and current unsatisfied therapeutic effects, as well as lack of practical application of BBR in sepsis, the nanostructured lipid carrier of BBR, more effective BBR derivatives, and drug combination strategies need to be investigated in various well-designed clinical trials in the future.

## Figures and Tables

**Table 1:** The protective effects and mechanisms of berberine in sepsis-related animal or cell model.

In vivo/vitro	Animal or cell model	Therapeutic dose	Effects and mechanisms	Reference
In vivo	Cecal slurry-induced neonatal sepsis in C57BL/6J mice (5-7 days)	50/100 *μ*g/mouse, i.p.	↑Survival rate; ↓intestinal injury; ↓IL-6, IL-1*β*, and TNF-*α*; ↑miR-132-3p; ↓FOXA1, p-I*κ*B*α*, and p65; and ↑I*κ*B*α*	[60]
In vivo	Mouse model of E. coli sepsis	5 mg/kg, i.p.	↑Survival rate; ↓IL-6, TNF-*α*, and CD4+ and CD8+ lymphocyte population in septic mice	[61]
In vivo	LPS/D-gal-induced C57BL/6 septic mouse model (6-8 weeks)	200 *μ*g/mouse, i.v.	↓Lung tissue injury; ↑survival rate; ↓IL-6, TNF-*α*, and IL-1*β*; (-) B cells%, T cells%, and subtypes of T cells in the spleen; and (-) CD11b + Gr-1+ cells in bone marrow cells	[30]
In vivo	LPS-induced male BALB/c mice (6-8 weeks)	50 mg/kg, i.g.	↓Ileum injury (injury score, intestinal villus height, gut mucosal weight, and intestinal permeability); ↓enterocyte apoptosis; and ↓TLR4 mRNA, p-I*κ*B*α*, MIP-2 production, and MPO activity	[18]
In vivo	CLP-induced male Wistar rats (260-300 g)	25/50/100 mg/kg/d, i.g.	↓Sepsis-related mortality; ↑hepatic and plasma ApoM level; ↓hyperglycemia, TNF-*α*, and IL-1*β*; and ↓sepsis-induced gluconeogenesis expression	[62]
In vivo	CLP-induced male Long-Evans rats (270-300 g)	25/50 mg/kg/d, i.p.	↓Microvascular permeability, serum endotoxemia levels	[17]
In vivo	CLP-induced male Wistar rats (270-310 g)	50 mg/kg/d, i.g.	↑Survival rates; ↓endotoxin levels and intestinal mucosal permeability; and ↑Zn^2+^ in intestinal mucosa, ZIP14 expression	[25]
In vivo	CLP-induced male SD rats (200-230 g)	50 mg/kg, i.g.	↓TNF-*α*, IL-6; ↓claudin-4, mucosal permeability; and ↓intestinal epithelial cell mortality	[12]
In vivo	CLP-induced SD rat (200-230 g)	50 mg/kg, i.g.	↓Intestinal epithelial cell death; ↓TLR2/4, TNF-*α*, and IL-6; and ↑zonula occludens-1	[20]
In vivo	Outer membrane vesicles (used for delivering LPS) challenged mice (25-30 g)	5 mg/kg	↓Vessel occlusion and fibrin deposition in liver microvasculature; ↓DIC markers (TAT and PAI-1); ↓multiorgan dysfunction and death rate; and ↓caspase-11-mediated coagulation activation	[50]
In vivo	LPS-induced male C57BL/6 mice (8 weeks)	10 mg/kg, injected into peritoneal cavity	↓Lung damage and injury scores; ↓injury of pulmonary permeability; ↓ROS level, IL-6/-18/-1*β*, and TNF-*α*; ↑IL-10; and ↓p-NF-*κ*B, Nlrp3	[31]
In vitro	HepG2 cells	5/10/20 *μ*M	↓TLR4 mRNA	[62]
In vitro	LPS or CpG-ODN-induced BMDM	5 *μ*M	↓IL-6, TNF-*α*, and IL-1*β*; ↓p-I*κ*B*α*/*β*, p-I*κ*B*α*, and NF-*κ*B nuclear translocation	[30]
In vitro	LPS-induced RIMECs	10/20 *μ*M	↓RIMEC permeability; ↑transendothelial electrical resistance; and ↑*β*-catenin, claudin-12, and VE-cadherin	[17]
In vitro	LPS-induced Caco-2 cells	20 *μ*M	↑Intracellular zinc content; ↑ZIP14, claudin-1, and occludin expression	[25]
In vitro	LPS-stimulated HPMECs	2.5 *μ*M	↓ROS level, IL-6/-18/-1*β*, and TNF-*α*; ↑IL-10; and ↓Nlrp3	[31]
In vitro	*E. coli*-stimulated primary macrophages and THP-1 cells	5 *μ*M	↓Cytotoxicity; ↓pyroptosis; and ↓caspase-11 pathway	[50]
In vitro	LPS-induced HUVECs	1.25/2.5/5 *μ*M	↓Endothelial glycocalyx degradation; ↓endothelial glycocalyx damage factors (ROS, heparanase, and MMP9)	[29]

ApoM: apolipoprotein M; BMDM: bone marrow-derived macrophages; CLP: ligation and puncture; D-gal: D-galactosamine; HUVECs: human umbilical endothelial cells; HPMECs: human pulmonary microvascular endothelial cells; i.g.: intragastric gavage; i.p.: intraperitoneal injection; i.v.: intravenous injection; RIMECs: rat intestinal microvascular endothelial cells; ROS: reactive oxygen species.

## Data Availability

The data used to support the review are included in the article.
